# Structural and Functional Brain Patterns of Non-Motor Syndromes in Parkinson’s Disease

**DOI:** 10.3389/fneur.2018.00138

**Published:** 2018-03-12

**Authors:** Tino Prell

**Affiliations:** ^1^Department of Neurology, Jena University Hospital, Jena, Germany

**Keywords:** Parkinson’s disease, cognition, imaging, positron emission tomography, pain, fatigue, impulsive behavior disorders

## Abstract

Parkinson’s disease (PD) is a common, progressive and multisystem neurodegenerative disorder characterized by motor and non-motor symptoms. Advanced magnetic resonance imaging, positron emission tomography, and functional magnetic resonance imaging can render the view toward understanding the neural basis of these non-motor syndromes, as they help to understand the underlying pathophysiological abnormalities. This review provides an up-to-date description of structural and functional brain alterations in patients with PD with cognitive deficits, visual hallucinations, fatigue, impulsive behavior disorders, sleep disorders, and pain.

## Introduction

Parkinson’s disease (PD) is a common and devastating, progressive movement disorder. Its hallmark pathology is the loss of dopaminergic neurons in the substantia nigra, causing the key motor symptoms of tremor, rigidity, and bradykinesia. However, due to its multisystem character the disease manifests with various non-motor symptoms (NMS), such as hyposmia, depression, cognitive decline, and psychosis, autonomic disturbances, and sleep disturbances. Advanced neuroimaging methods render the view toward understanding the neural basis of these NMS in PD, as they allow a “window” into the underlying pathophysiological abnormalities. This review provides an up-to-date description of structural and functional alterations assessed by magnetic resonance imaging (MRI), positron emission tomography (PET), and functional magnetic resonance imaging (fMRI) underlying distinct NMS in the brain of PD patients.

**Figure 1:**
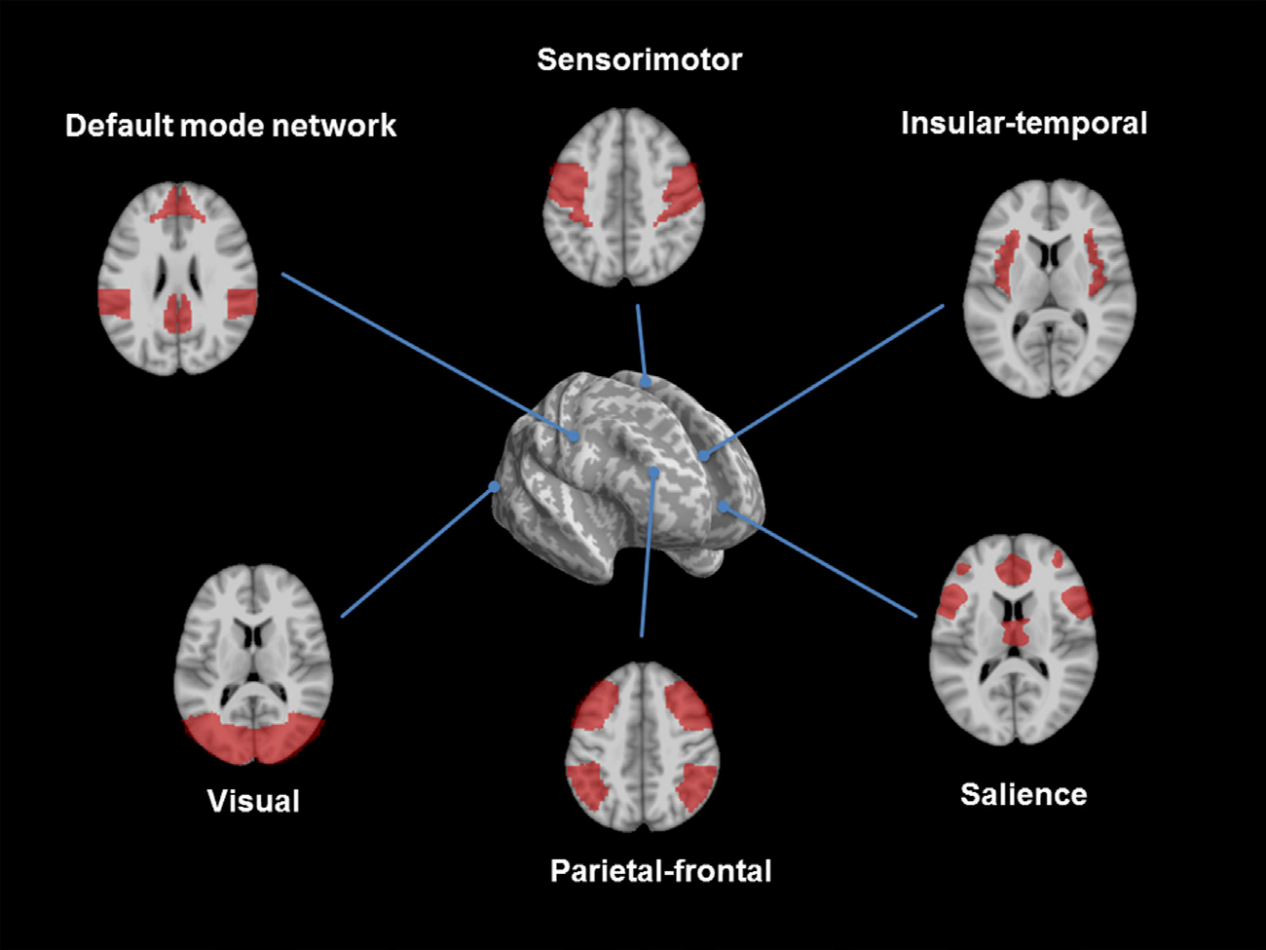
Overview of resting-state networks. The figure shows the consistent reported resting-state networks: the default mode network, the sensorimotor, the insular/temporal and anterior cingulate cortex regions, the salience, the executive control, and the visual network (figures developed with SPM 8, www.fil.ion.ucl.ac.uk/spm).

Structural changes can be studied by using advanced MRI methods, such as *v*oxel-based morphometry (VBM), diffusion tensor imaging (DTI), or susceptibility weighted imaging (SWI). The VBM is based on 3D-T1-weighted images and compares changes of gray and white matter volume among groups. DTI and fiber tracking are useful tools to study the three-dimensional diffusion of water as a function of spatial location and to display microstructural changes of white matter. The diffusion tensor may be used to characterize the magnitude, anisotropy, and orientation of the diffusion tensor. SWI is particularly sensitive to compounds which distort the local magnetic field, such as venous blood, hemorrhage, iron, and calcium. All these methods can be applied without any *a priori* assumptions (whole-brain approach) or with respect to a region of interest. The fMRI measures brain activity by detecting changes associated with blood flow because cerebral blood flow and neuronal activation are coupled [blood oxygen level-dependent contrast imaging (BOLD)]. The resting-state fMRI (rs-fMRI) evaluates regional interactions that occur when a subject is not performing an explicit task. The resting-state functional connectivity technique investigates the correlation patterns of BOLD signals between regions of interest and other brain regions. There are several anatomically separated brain regions that are functionally linked during rest: the default mode network (DMN), the sensorimotor, visual, executive (mainly dorsolateral prefrontal cortex and anterior cingulate cortex), salience (insular), frontal, parietal, auditory, and temporal networks (Figure 1). Functional communication between these brain regions plays a pivotal role in complex cognitive processes. Thus, the examination of functional connectivity provides insights in the core organization of the brain. There are several methods to analyze rs-fMRI data. Overall, one can distinguish between model-dependent and model-free methods. Model-dependent methods, including seed methods, correlate the data of a specific brain region (the “seed”) against the time-series of other regions. Model-free methods include for instance the principal component analysis or independent component analysis. Here, connectivity patterns were analyzed without the need of defining an *a priori* seed region (1).

An important issue in analyzing structural and functional MRI data is the specification of an appropriate threshold for statistical maps. Running the statistical analysis separately for each voxel creates the problem of multiple comparisons which increases the risk of false-positive results. Therefore, especially whole-brain and model-free analyses should incorporate a correction for multiple comparisons, such as the family-wise error method. The PET is a nuclear functional imaging technique that detects pairs of gamma rays emitted indirectly by a positron-emitting radionuclide (tracer). Depending on the research or clinical questions several tracers are available [e.g., fluorine-18 (F-18) fluorodeoxyglucose (FDG)].

## Search Strategies and Selection Criteria

PubMed database was searched for articles on neuroimaging studies in PD by using a number of terms and combinations (“Parkinson,” “functional imaging,” “PET,” “MRI,” “non-motor,” “fatigue,” “hallucination,” “dementia,” “cognition,” “pain,” “impulsive compulsive behavior,” “impulse control disorders,” “sleep disorders,” “RBD”). Articles were restricted to those: (1) in English and (2) published between 2000 and November, 2017. All abstracts were screened for relevance and the most pertinent articles were reviewed in full, with further examination of the corresponding reference lists. Since imaging of apathy, anxiety, and depression were recently reviewed these NMS were excluded (2).

## Cognition

**Figure 2:**
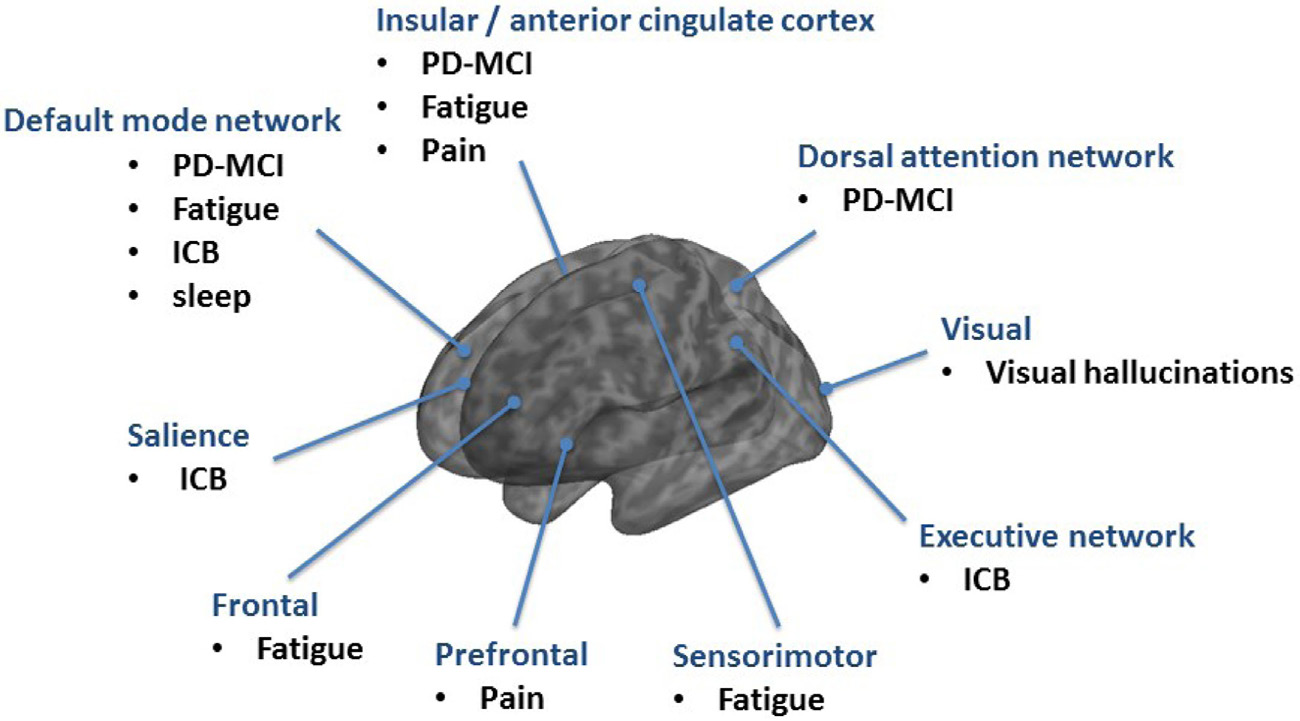
Overview of resting-state networks mainly involved in non-motor syndromes in Parkinson’s disease (PD).

Cognitive deficits are common in PD and can be present even as mild dysfunction in the prodromal and early stages or as dementia (PDD) in advanced stages (3). Cognitive function in PD deteriorates over time. 24% of the PD patients have cognitive disturbances at onset of the disease and every second patient shows progressive cognitive decline in the first 3 years (4, 5). In the long-term follow-up, the cumulative prevalence rates of PDD increase up to 80%. Therefore, it seems that, regardless of the time of PD onset, the evolution of PDD occurs at around 70 years of age, and affects cognitive domains in a similar way (6, 7). There is still discussion about the role and localization of mild cognitive impairment in PD (PD-MCI) in between the spectrum of cognitive function and dysfunction. Therefore, the Movement Disorders Society Study group developed PD-MCI criteria (8). PD-MCI is characterized by a decreased prefrontal, temporal and parietal metabolism as well as an increase in brainstem/cerebellar metabolism in FDG-PET (9, 10). It seems that with ongoing cognitive decline in PDD this hypometabolism spreads to the anterior cingulate cortex (10). However, the analysis of only 13 patients with PDD and the missing correction for multiple comparisons in the study by Yong et al. limits the generalizability of this result (10). The anterior cingulate cortex plays a role in a wide variety of autonomic functions and certain higher-level functions, such as decision-making, impulse control, and error detection which explains typical clinical problems in PDD. A recent fMRI study shows that a hypoactivation in the anterior cingulate cortex can also be observed in early and non-demented PD which could probably explain the impaired ability to shift attention between stimuli (i.e., shifting attentional “set”) as part of the dysexecutive syndrome in PD (11). A complex network analysis approach revealed that PD-MCI is characterized by both, increments of local interconnectedness and connectivity decrements predominantly affecting long-range connections (12). Of notice, hyperconnectivity was demonstrated in PD patients without cognitive deficits, suggesting a recruitment of additional resource areas as initial response to progressive cell loss (13). However, with ongoing cognitive decline hyoconnectivites and disruptions of networks occur. In particular, the DMN emerged as a key function for cognitive deficits in PD (14). A decreased functional connectivity of the medial temporal lobe and inferior parietal cortex was found within the DMN (15). Using fMRI, a dysfunction of DMN during an executive task was detected in PD (16) and it was shown that the posterior cingulate cortex as major node within the DMN is linked to cognitive impairment in PD (17, 18). Using the posterior cingulate cortex as a seed for analysis, a significant decrease of connectivity was found in PD-MCI in the bilateral prefrontal cortex, left parieto-occipital junction, and right temporal gyrus and in PDD in the right inferior frontal gyrus as compared to non-demented PD (19, 20). A model-free approach using independent component analysis revealed that the connectivity between the dorsal attention network and right anterior insula with its adjacent frontal areas is reduced in PD-MCI and increased between posterior cortical regions and the DMN (21). The anterior insula is, therefore, critical for switching between dorsal attention network and DMN in the resting state and across different tasks (21, 22) (Figure 2).

Positron emission tomography studies using [^11^C]-MP4A showed a significantly reduced cortical acetylcholinesterase activity in PDD and PD subjects when compared with healthy control subjects (23). The reduced cortical binding of [^11^C]-MP4A with increasing signal diminution from frontal to occipital regions in PDD was comparable to the pattern in dementia with Lewy bodies (24). Whereas, in Alzheimer’s disease the reduction of [^11^C]-MP4A binding is restricted to the hippocampus, the temporoparietal cortex, and amygdala which underlines that PDD relies on an extensive cholinergic denervation (25, 26).

In terms of structural changes, there are conflicting results in PD, PD-MCI, and PDD. One study observed gray matter loss in cortical and subcortical regions of the prefrontal, temporal, and parieto-occipital cortex in non-demented PD patients in comparison to healthy controls, while another did not (27, 28). A meta-analysis of gray matter volume differences between patients with PD and healthy controls mainly found that patients with PD have regional gray matter volume reductions in the left inferior frontal gyrus extending to the superior temporal gyrus and the insula (29). This is of interest, since the left inferior frontal gyrus is specifically associated with cognitive processes and is involved in processing the motivational or emotional value of incoming information (29). Studies investigating gray matter atrophy in PD-MCI were negative or observed gray matter atrophy in the frontal, hippocampal, temporal, and parieto-occipital regions (28, 30–38). Structural changes in frontal and limbic system in PD-MCI were found to be associated with impaired performance in the Mini-Mental State Examination score, whereas frontal lobe atrophy was found to be associated with low performance in the Montreal Cognitive Assessment score, suggesting that atrophy of limbic lobes is associated with impaired memory, whereas frontal lobe atrophy is associated with executive dysfunction (39). The conflicting results in terms of PD-MCI may reflect the limitations of VBM to track subtle cortical atrophy in early stages of the disease, because cortical thinning assessed by surfaced-based analyses was consistently observed in frontal, temporal, and parietal regions in PD-MCI (40–43). While the extent of gray matter atrophy in non-demented PD and PD-MCI is a topic of contentious debate, the gray matter atrophy is well established in PDD. Subsequently, with disease progression and onset of PDD, diffuse gray matter loss becomes evident bilaterally in the hippocampus and parahippocampal gyrus, and in the occipital lobe, the frontal and parietal lobe, as well as some subcortical regions (31, 44).

Beside gray matter atrophy, the analysis of whiter matter with DTI revealed widespread changes in PD patients with cognitive dysfunction. Relative to healthy controls and non-demented PD patients, PD-MCI patients showed widespread white matter abnormalities in the cingulum, anterior and superior corona radiata, genu, and body of the corpus callosum, and inferior fronto-occipital, uncinate, and superior longitudinal fasciculi (37, 45, 46). The loss of microstructural white matter integrity in PD increases with cognitive dysfunction (45). These DTI results indicate that central white matter tracts degenerate early in PD and that cognitive dysfunction in PD-MCI is linked to axonal damage (46).

These results have expanded the understanding of cognitive impairment in PD beyond fronto-striatal circuit dopaminergic deficits. Structural MRI studies have revealed gray matter atrophy and disruptions of white matter integrity in PDD, although findings in non-demented PD and PD-MCI remain inconstant. The lack of reproducibility in PD-MCI and non-demented PD can be explained by the heterogeneity of subjects, low sample size in some studies, different types of analysis in terms of methods, MRI parameters, or template choice. Furthermore, there are opposing views on PD-MCI itself and the neuropsychological assessments differ widely between the studies. In addition, there is a large heterogeneity across studies in terms of clinical stage, medication, and presence of other NMS. In particular, there is a need for multicenter longitudinal studies to clarify the spatial and temporal progression of morphological changes in cognitive decline in PD.

## Fatigue

Fatigue is one of the most common and disabling NMS in PD (47–49). Fatigue is a leading cause of disability and dramatically impairs quality of life (50, 51). Although a lack of consensus exists regarding a precise definition of PD fatigue, the core of fatigue is a “feeling of abnormal and overwhelming tiredness and lack of energy, distinct both qualitatively and quantitatively from normal tiredness” [cited from Ref. (52), p. 54]. Despite its enormous impact and high prevalence, little progress has been made in understanding the etiology or pathophysiology of fatigue.

The rs-fMRI demonstrates that brain areas, including frontal, postcentral, and anterior cingulate cortex regions, are involved in fatigue in PD patients (53). In another rs-fMRI study the amplitude of low-frequency fluctuations (ALFF) was used to measure regional brain activity and functional connectivity was investigated at a network level. PD-related fatigue was associated with ALFF changes in the attention network (right middle frontal gyrus) and in the salience network (left insula, right midcingulate cortex). Corresponding to that, functional connectivity was altered mainly in the temporal, parietal, and motor cortices (54). The study by Tessitore et al. studied a cohort of “drug-naïve” patients with PD with rs-fMRI and independent component analysis as model-free approach (55, 56). Fatigue was associated with a decreased connectivity in the sensorimotor network (supplementary motor area) and an increased connectivity in the DMN (prefrontal and posterior cingulate cortices). Fatigue severity, as assessed with the 16-item Parkinson fatigue scale was correlated with both sensorimotor and DMN connectivity changes (55, 56). The involvement of the frontal lobe in PD-fatigue was also demonstrated with SPECT (99mTc-HMPAO) showing a significant correlation between the fatigue scale and the reduction of perfusion in the frontal lobe in patients with PD (57). In a recent FDG-PET study high Fatigue Severity Scale scores were associated with brain hypermetabolism in areas, including the right middle temporal gyrus (Brodmann area 37) and left middle occipital gyrus (Brodmann area 19), and hypometabolism in regions, such as the right precuneus (Brodmann area 23), left inferior frontal gyrus (Brodmann area 45), and left superior frontal gyrus (orbital part, Brodmann area 11) (54). The FDG-PET revealed that metabolic changes in cortical regions associated with the salience (i.e., right insular region) and default (i.e., bilateral posterior cingulate cortex) networks display a significant correlation with the level of fatigue (58). In terms of structural changes, there were no significant volume differences of gray matter between PD with and without fatigue in the VBM analysis (55, 56). In summary, multiple brain areas underlie fatigue, including frontal, temporal, and parietal regions indicative of emotion, motivation, and cognitive functions (Figure 2).

## Visual Hallucinations

Visual hallucinations are the most common manifestation of psychosis in PD and are predictive for a rapid cognitive decline. VBM studies have reported gray matter atrophy in multiple regions, but, overall, the results remain inconsistent. Gray matter atrophy was observed in areas related to visuospatial processing, attention, and memory, suggesting visual hallucinations are the correlate of dysfunctions in multiple cortical as well as subcortical brain regions. The involved areas include the primary visual cortex, visual association cortex, limbic and para-limbic regions, the pedunculopontine nucleus, and substantia innominate (59–65).

Also white matter changes were found when visual hallucinations are present. The mean diffusivity was increased in the parieto-temporal region in the non-demented PD patients with visual hallucinations and increased diffusely in the presence of dementia, including the fronto-occipital regions (66). Functional imaging studies consistently revealed the involvement of visual pathways in the pathogenesis of visual hallucinations, including alterations in both dorsal and ventral visual pathways (67–74). It was hypothesized that PD patients with visual hallucinations have a reduced responsiveness to external visual stimuli (bottom-up) and an abnormally increased frontal activation (top-down) (67, 75). Regional cerebral blow flow, assessed with SPECT and N-isopropyl-p-[(123)I]iodoamphetamine, showed a hypoperfusion of the right fusiform gyrus (visual recognition) and an hyperperfusion of the right superior and temporal gyri (generation of complex visual images) in PD patients with visual hallucinations (75). Also the serotonergic system seems to play a role in visual hallucinations. Patients having PD with visual hallucinations demonstrate increased serotonin 2A receptor binding (PET with [^18^F]-Setoperone) in the ventral visual pathway (including the bilateral inferooccipital gyrus, right fusiform gyrus, and inferotemporal cortex) as well as the bilateral dorsolateral prefrontal cortex, medial orbitofrontal cortex, and insula (76).

## Impulse Control Behavior

Impulsive control behaviors (ICB) include gambling disorder, binge eating disorder, compulsive sexual behavior, and compulsive shopping and occur in 17% of patients treated with a dopamine agonist. These behaviors probably reflect the interactions of the individual’s susceptibility and the dopaminergic medicationsin PD (77). There is some evidence that some patients are prone to develop ICB. In general, PD-ICB has been associated mainly with brain alterations involving the fronto-striatal and fronto-limbic circuits. In a recent study in PD patients with and without ICB, the presence of ICB symptoms was associated with an increased connectivity within the salience network and DMN, as well as with a decreased connectivity within the central executive network (78). Drug-naïve PD patients who develop ICB in the follow-up showed at baseline a decreased connectivity in the DMN and executive network and increased connectivity in the salience network, suggesting that these cognitive and limbic connectivity changes are predictive for the development of ICB in PD (79). The involvement of the dopaminergic system in the pathophysiology of ICB has been demonstrated by several functional imaging studies showing an abnormal sensitization of the dopaminergic system. In line with the sensitization theory PET studies using [^11^C]-Raclopride have shown a greater striatal dopamine release after levodopa intake in PD patients with ICD. In contrast to PD without ICB the PD patients with dopamine dysregulation syndrome exhibited enhanced levodopa-induced ventral striatal dopamine release and this sensitized striatal dopamine transmission correlated with self-reported compulsive drug “wanting” but not “liking” (80). Also a reduced [^11^C]-Raclopride binding in the ventral striatum of PD patients with pathological gambling likely reflects greater dopaminergic release (81). Interestingly that greater striatal dopamine release and abnormal sensitization of the dopaminergic system after a levodopa dose intake was only observed following reward-related cue exposure, relative to neutral cue exposure (82). This heightened response of striatal reward circuitry to heterogeneous reward-related visual cues was observed among a group of patients with different kind of ICBs. Therefore, one can assume that in vulnerable individuals there is some kind of global sensitization to appetitive behaviors with dopaminergic therapy (82). In a fMRI study of PD patients with hypersexuality the exposure to sexual cues significantly increased sexual desire and this was accompanied by significant signal changes in regions corresponding to emotional, cognitive, autonomic, visual, and motivational processes (limbic, paralimbic, temporal, occipital, somatosensory, and prefrontal cortices). Moreover, the increased sexual desire correlated with enhanced activations in the ventral striatum, and cingulate and orbitofrontal cortices (83). When the PD patients with hypersexuality were OFF medication, the activation during the presentation of sexual cues decreased. However, this decrease was not present when the patients were ON medication. One can, therefore, hypothesize that dopamine drugs may release inhibition within local cortical neuronal circuits (83). In summary, a consistent feature of ICB is the increased cue reactivity in the striatum. Using a pharmacological manipulation and a risk taking task while performing fMRI PD patients with ICB made more risky choices. This was accompanied with decreased activity in the orbitofrontal cortex and anterior cingulate cortex in comparison to PD controls (84). PD patients with ICB appear to have a bias toward risky choices and dopamine agonists seem to enhance the sensitivity to risk because they impair the striatal risk evaluation (84).

Furthermore, PD patients with pathological gambling showed resting-state overactivity in the orbitofrontal cortex, the hippocampus, the amygdala, the insula, and the ventral pallidum (mesocorticolimbic network) (85). The mesocorticolimbic dopamine network guides reward-motivated behavior; however, its role in PD-ICB is not yet understood. A recent fMRI study showed elevated ventral striatal connectivity to the anterior cingulate gyrus, orbitofrontal cortex, insula, putamen, globus pallidus, and thalamus in PD patients with ICB, suggesting that PD-ICB patients have elevated network connectivity in the mesocorticolimbic network. Behaviorally, proficient reward-based learning is related to this enhanced limbic and ventral striatal connectivity (86) (Figure 2).

In terms of gray matter changes, the studies in PD-ICB are inconsistent (55, 56, 87, 88). One study aimed to answer the issue mentioned above whether *de novo* PD patient who develop ICB have specific structural abnormalities. Patients who went on to develop ICB did not show significant structural changes in comparison to PD patients without ICB (89). Reduced cortical thickness of fronto-striatal regions but also increased volume of amygdala and a positive correlation between ICB severity and fronto-parietal gray matter volumes has been reported (90, 91). In terms of white matter structural changes, an increased fractional anisotropy of the genu of the anterior corpus callosum, and right internal capsule, right posterior cingulum, and right thalamic radiations was observed in PD-ICB (92). A recent comprehensive study using DTI, rs-fMRI, and surface-based morphometry provided a broad picture of structural and functional alterations in PD-ICB patients. Compared with PD without ICB, patients with PD-ICB were characterized by precentral and superior frontal cortical thinning, and motor and extramotor white matter tract damage. Relative to PD without ICB, PD-ICB patients were characterized by a more severe involvement of frontal, meso-limbic, and motor circuits. ICB in PD is then probably the result of a disconnection between sensorimotor, associative, and cognitive networks (93).

## Sleep Disorders

Sleep disorders in PD are common and include heterogeneous group of symptoms and reasons, such as nightly motor symptoms, REM sleep behavior disorders (RBD), or daytime sleepiness. RBD is a parasomnia with dream enactment and violent limb moves, which can occur in the prodromal phase of PD. Sleep disorders were found to be related to nigrostriatal dopaminergic degeneration and around 20–40% of RBD patients in the prodromal state of PD show deficits in the dopamine transporters (94, 95). The results in terms of brain perfusion and metabolism in prodromal cohorts, however, remain inconsistent [recently reviewed in Ref. (96, 97)]. Since sleep can have a restorative role in the brain a major question in PD is the link between sleep disturbances and cognitive deficits. PD patients with sleep disturbance showed poorer performance in attention/working memory and were characterized by a more extensive cortical thinning in the left fronto-parietal regions and white matter disintegration in widespread regions when compared to those without sleep disturbance (98). Cortical functional connectivity in PD patients with sleep disturbance with a seed in the DMN and dorsal attention network exhibited a less severe decrease when compared to those without sleep disturbance. These data suggest that sleep disturbances are associated with white matter and alterations in functional networks in conjunction with cognitive impairment (98). In PD patients with RBD, whiter matter alterations were also observed in the cingulum and left inferior occipital fasciculus and might explain faster cognitive decline in terms of visual recognition and visuospatial dysfunction and executive function in these patients (99, 100). This is in line with the fact that RBD is a predictor of PDD.

## Pain

Pain is a frequent symptom in PD with great impact on mobility and quality of life. Pain can occur in both premotor and motor stages and may be linked to musculoskeletal, dystonic, radicular, neuropathic and central causes (101, 102). The so called “pain matrix” as a fluid system composed of several interacting networks encompasses several brain areas which are involved in pain processing (103, 104). The main components of this network in terms of acute pain are: primary and secondary somatosensory, insular, anterior cingulate, prefrontal cortices, and thalamus (105). However, pain is not only nociception because many factors (e.g., cognition, depression, and social status) can separately influence pain perception. Nociceptive inputs can activate complex interactions among central sites, including cortical regions that are active in cognitive, emotional, and reward functions (106). Therefore, multiple other areas were found to be related to pain processing, such as basal ganglia, brainstem, cerebellum, and hippocampus (103, 106, 107). With ongoing pain or chronic pain, there is a continuous reorganization of the cortex (108). The pain threshold in PD patients was found to be lower than in healthy controls and the administration of levodopa significantly raised the pain threshold in PD but not in controls. With the reduced pain threshold, there was a significant increase in pain-induced activation in the pain-matrix, namely right insula and prefrontal and left anterior cingulate cortices in PD compared to control group. Moreover, levodopa significantly reduced this pain-induced cortical activation and increases pain threshold, providing evidence for the involvement of the dopaminergic system in the modulation of pain in PS (109, 110). In early, “drug-naive” PD patients not experiencing pain symptoms, the event-related fMRI suggest that a functional remodulation of pain processing pathways occurs even in the absence of clinically overt pain symptoms. A greater activation of the left somatosensory cortex, left cerebellum and right low pons (in an area encompassing the nucleus rafe magnus and the gigantocellular/paragigantocellular nuclei) during noxious stimulations is present in “drug-naive” PD patients when compared to healthy controls (111). In summary, these data suggest that a compensatory reorganization of pain-related brain areas, induced by early neuropathological changes, is present in “drug-naive” PD patients, not reporting pain symptoms. These mechanisms may then become dysfunctional during disease course and impact pain-threshold and contribute to the emergence of pain symptoms in more advanced PD stages (111).

## Conclusion

The multisystem character of PD and its clinical heterogeneity in disease presentation and progression challenges the understanding of NMS in PD, especially at early stages. To better understand the nature and pathophysiology of NMS multiple biomarkers derived from different imaging modalities seems to be the appropriate way. Moreover, ideally these data should be linked to other biomarkers, e.g., derived from cerebrospinal fluid in order improve our understanding and to weight the importance of imaging findings. Further research is needed to perform multicenter studies, to improve measurement techniques, and to standardize research protocols. An important issue in PD is that patients usually have a large panel of different motor and NMS with each having impact on the individual brain signatures. Therefore, it is of importance to assess as many as possible cofounders with valid and reliable methods before analyzing imaging data. This is of importance, because future clinical decision-making and treatment may likely rely upon *in vivo* imaging.

## Author Contributions

Developing and writing: TP.

## Conflict of Interest Statement

The author declares that the research was conducted in the absence of any commercial or financial relationships that could be construed as a potential conflict of interest. The reviewer MG and handling Editor declared their shared affiliation.
